# Supplementary material for: Mineral reactivity determines root effects on soil organic carbon
Source: Nat Commun. 2023 Aug 16;14:4962. doi: 10.1038/s41467-023-40768-y (PMC10432558; doi:10.1038/s41467-023-40768-y)
Supplement: Supplementary file 1 — Supplemental Information File [file 41467_2023_40768_MOESM1_ESM.pdf]

Supplementary Information

Mineral reactivity determines root effects on soil organic carbon

Guopeng Liang[1,2], John Stark[1], Bonnie Grace Waring[1,3*]

[1] Department of Biology, Utah State University, Logan UT USA 84322

[2] Present address: Department of Forest Resources, University of Minnesota, Saint Paul, MN 55108

[3] Present address: Grantham Institute on Climate Change and the Environment and Georgina Mace Centre for the Living Planet, Imperial College London, London UK

*Author for correspondence: bonnie.waring@gmail.com

Table S1a. Two-sided analysis of variance examining effects of mineral reactivity, inoculum type, and presence of root exudates on artificial soil parameters during first phase of the experiment (1 – 3 months). Values shown are F statistics, with *P* values in parentheses. **b.** Carbon use efficiencies (unitless) in each treatment group (mean ± 1 SE). Other responses are visualized in Fig S1.

a.

	**Numerator df**	**Total C pool***	**Cumulative CO_2**	**Microbial biomass**	**C use efficiency**	**Bacterial richness**	**Fungal richness**
		g C	g CO_2-C g^{-1}	μg g^{-1}	unitless	No. ASVs	No. ASVs
Mineral reactivity	2	2.64 (0.080)	2.64 (0.080)	**3.42** (0.039)	**4.90** (0.011)	**114.3** (<0.001)	**9.08** (<0.001)
Inoculum	1	**9.33 (0.003)**	**9.33** (0.003)	0.10 (0.756)	**4.67** (0.035)	**153.5** (<0.001)	**360.9** (<0.001)
Root exudates	1	**6.81 (0.011)**	0.69 (0.408)	**13.58** (<0.001)	0.45 (0.506)	0.06 (0.811)	0.82 (0.369)
Mineral × inoculum	2	0.64 (0.527)	0.65 (0.527)	1.32 (0.275)	0.71 (0.496)	**34.78** (<0.001)	1.16 (0.319)
Mineral × root	2	0.70 (0.502)	0.70 (0.502)	**8.54** (<0.001)	0.50 (0.607)	2.55 (0.088)	0.41 (0.668)
Inoculum × root	1	0.97 (0.329)	0.97 (0.328)	1.10 (0.298)	**4.96** (0.030)	2.91 (0.094)	0.56 (0.460)
Mineral × inoculum × root	2	0.04 (0.961)	0.04 (0.961)	1.41 (0.253)	2.39 (0.101)	2.90 (0.064)	1.1 (0.340)

*calculated via a mass-balance approach at the 3-month harvest

b.

**Mineral treatment**	**Inoculum treatment**	**Root treatment**	**CUE**
Kaolinite	Bacteria-only	Artificial roots	0.665 ± 0.029
		Control	0.740 ± 0.018
	Fungi and bacteria	Artificial roots	0.692 ± 0.018
		Control	0.616 ± 0.041
Montmorillonite	Bacteria-only	Artificial roots	0.726 ± 0.020
		Control	0.770 ± 0.011
	Fungi and bacteria	Artificial roots	0.705 ± 0.010
		Control	0.721 ± 0.009
Goethite	Bacteria-only	Artificial roots	0.720 ± 0.013
		Control	0.725 ± 0.016
	Fungi and bacteria	Artificial roots	0.718 ± 0.017
		Control	0.711 ± 0.018

Table S2. Permutational analysis of variance (PERMANOVA) examining effects of mineral reactivity, inoculum type, and presence of root exudates on bacterial and fungal community composition (identity and relative abundance of amplicon sequence variants) in the first phase of the experiment (1 – 3 months), for artificial soils only. Values shown are F statistics, with *P* values in parentheses

	**Bacteria**		**Fungi**	
	F (P value)	R^2	*F (P value)*	R^2
Mineral reactivity	**19.70** (<0.001)	0.292	**9.57** (<0.001)	0.161
Inoculum	**18.48** (<0.001)	0.137	**27.80** (<0.001)	0.233
Root exudates	**2.80** (0.029)	0.021	**2.72** (0.037)	0.022
Mineral × inoculum	**4.70** (<0.001)	0.070	**3.30** (0.002)	0.055
Mineral × root	**3.13** (0.006)	0.046	1.54 (0.114)	0.026
Inoculum × root	**2.61** (0.039)	0.019	0.70 (0.584)	0.005
Mineral × inoculum × root	**2.06** (0.039)	0.031	2.09 (0.062)	0.035

Table S3. Two-sided analysis of variance examining effects of mineral reactivity, inoculum type, aboveground carbon inputs, presence of root exudates, and date of microcosm harvest (month 7 vs 13) on artificial soil parameters in the second phase of the experiment (months 4 – 13). Values shown are F statistics, with *P* values in parentheses

	**Numerator df**	**Cumulative CO_2**	**Microbial biomass**	**Mineral-associated organic C (MAOC)**	**Bacterial species richness**	**Fungal species richness**
		g CO_2-C g^{-1}	μg g^{-1}	g g^{-1}	No. ASVs	No. ASVs
Mineral reactivity (M)	2	**18.84** (<0.001)	**3.27** (0.041)	**12.85** (<0.001)	**62.85** (<0.001)	**30.76** (<0.001)
Inoculum (I)	1	0.06 (0.803)	**8.82** (0.004)	**25.30** (<0.001)	**197.23** (<0.001)	**1164.86** (<0.001)
Root exudates (R)	1	**125.25** (<0.001)	**16.89** (<0.001)	**26.49** (<0.001)	**115.24** (<0.001)	**41.94** (<0.001)
Aboveground carbon inputs (C)	2	**89.33** (<0.001)	**4.46** (0.013)	0.65 (0.521)	**7.52** (<0.001)	**12.52** (<0.001)
Harvest (H)	1	**1075.46** (<0.001)	**294.32** (<0.001)	**103.61** (<0.001)	**16.32** (<0.001)	**7.26** (0.008)
M × I	2	**4.96** (0.008)	2.56 (0.081)	0.02 (0.979)	**15.72** (<0.001)	**3.96** (0.021)
M × R	2	**7.15** (0.001)	**5.12** (0.007)	**20.39** (<0.001)	**20.34** (<0.001)	2.21 (0.113)
I × R	1	0.40 (0.529)	0.24 (0.622)	**13.23** (<0.001)	**13.01** (<0.001)	0.57 (0.453)
M × C	4	**2.65** (0.036)	1.86 (0.121)	1.25 (0.291)	**3.91** (0.005)	1.94 (0.108)
I × C	2	0.11 (0.894)	0.08 (0.919)	**3.06** (0.050)	**4.30** (0.016)	**3.44** (0.035)
R × C	2	1.65 (0.197)	0.11 (0.892)	**6.18** (0.003)	**6.83** (0.002)	**7.43** (<0.001)
M × H	2	**5.04** (0.008)	**17.17** (<0.001)	1.96 (0.145)	**3.16** (0.045)	2.12 (0.123)

$I \times H$	1	**4.52** (0.035)	0.12 (0.735)	**7.05** (0.009)	0.99 (0.321)	1.87 (0.173)
$R \times H$	1	**28.99** (<0.001)	3.70 (0.056)	2.61 (0.108)	**26.42** (<0.001)	0.34 (0.563)
$C \times H$	2	**10.64** (<0.001)	2.28 (0.106)	0.17 (0.844)	0.76 (0.472)	1.44 (0.241)
$M \times I \times R$	2	0.66 (0.518)	1.76 (0.176)	**5.48** (0.005)	**26.51** (<0.001)	0.01 (0.993)
$M \times I \times C$	4	1.47 (0.214)	0.74 (0.563)	2.34 (0.058)	**5.48** (<0.001)	1.23 (0.303)
$M \times R \times C$	4	0.75 (0.558)	**2.65** (0.036)	**7.17** (<0.001)	**5.47** (<0.001)	2.01 (0.096)
$I \times R \times C$	2	0.94 (0.394)	**3.97** (0.021)	2.21 (0.113)	**6.88** (0.001)	**3.80** (0.025)
$M \times I \times H$	2	0.84 (0.432)	0.46 (0.636)	0.86 (0.426)	**4.44** (0.024)	0.05 (0.948)
$M \times R \times H$	2	1.34 (0.266)	0.70 (0.497)	**19.44** (<0.001)	2.34 (0.100)	0.42 (0.656)
$I \times R \times H$	1	3.46 (0.065)	**5.49** (0.021)	1.03 (0.311)	2.66 (0.105)	0.00 (0.946)
$M \times C \times H$	4	1.04 (0.388)	**3.24** (0.014)	1.71 (0.152)	1.21 (0.308)	0.04 (0.006)
$I \times C \times H$	2	2.18 (0.117)	1.52 (0.223)	**5.25** (0.006)	0.23 (0.796)	2.52 (0.084)
$R \times C \times H$	2	0.21 (0.811)	1.07 (0.345)	1.62 (0.202)	1.15 (0.321)	0.94 (0.394)
$M \times I \times R \times C$	4	0.90 (0.466)	2.16 (0.076)	1.57 (0.186)	**5.61** (<0.001)	**4.52** (<0.001)
$M \times I \times R \times H$	2	0.20 (0.819)	1.17 (0.313)	**3.86** (0.023)	1.43 (0.242)	1.28 (0.281)
$M \times I \times C \times H$	4	0.32 (0.862)	1.66 (0.162)	**4.08** (0.004)	1.64 (0.168)	1.01 (0.403)
$M \times R \times C \times H$	4	0.98 (0.420)	1.01 (0.406)	0.80 (0.524)	0.92 (0.454)	2.43 (0.051)
$I \times R \times C \times H$	2	2.04 (0.134)	2.74 (0.068)	**5.14** (0.007)	0.92 (0.403)	0.88 (0.419)
$M \times I \times R \times C \times H$	4	0.21 (0.931)	1.72 (0.148)	0.40 (0.807)	1.02 (0.400)	0.49 (0.740)

Table S4. Permutational analysis of variance (PERMANOVA) examining effects of mineral reactivity, inoculum type, aboveground carbon inputs, presence of root exudates, and date of microcosm harvest on bacterial and fungal community composition (identity and relative abundance of amplicon sequence variants) in artificial soils during the second phase of the experiment (months 4 – 13).

	**Bacteria**		**Fungi**	
	F (*P*)	R^2	F (*P*)	R^2
Mineral reactivity (M)	**44.95** (<0.001)	0.183	**32.22** (<0.001)	0.159
Inoculum (I)	**39.33** (<0.001)	0.080	**78.93** (<0.001)	0.195
Root exudates (R)	**50.33** (<0.001)	0.102	**7.52** (<0.001)	0.019
Aboveground carbon inputs (C)	**4.61** (<0.001)	0.019	**2.66** (0.006)	0.013
Harvest (H)	**11.96** (<0.001)	0.024	**9.58** (<0.001)	0.024
M × I	**7.21** (<0.001)	0.029	**9.89** (<0.001)	0.049
M × R	**7.38** (<0.001)	0.030	**3.94** (<0.001)	0.019
I × R	**3.65** (0.011)	0.007	**2.68** (0.016)	0.006
M × C	1.42 (0.086)	0.012	1.22 (0.189)	0.012
I × C	**2.03** (0.027)	0.008	**1.79** (0.048)	0.009
R × C	**2.95** (0.003)	0.012	1.26 (0.246)	0.006
M × H	**2.32 (0.012)**	0.009	1.07 (0.354)	0.005
I × H	**3.20 (0.009)**	0.007	1.20 (0.261)	0.003
R × H	**8.04 (0.001)**	0.016	2.05 (0.061)	0.005
C × H	1.18 (0.268)	0.005	1.55 (0.096)	0.008
M × I × R	**7.01 (0.001)**	0.028	**1.96 (0.041)**	0.010

$M \times I \times C$	**1.62 (0.044)**	0.013	**1.91 (0.010)**	0.019
$M \times R \times C$	**2.21 (0.003)**	0.018	**1.79 (0.022)**	0.018
$I \times R \times C$	**1.79 (0.041)**	0.007	1.05 (0.393)	0.005
$M \times I \times H$	**2.74 (0.002)**	0.011	1.38 (0.146)	0.007
$M \times R \times H$	**4.01 (0.001)**	0.016	**1.91 (0.040)**	0.009
$I \times R \times H$	**7.80 (0.001)**	0.016	**2.62 (0.019)**	0.006
$M \times C \times H$	1.16 (0.259)	0.009	1.02 (0.428)	0.010
$I \times C \times H$	1.05 (0.347)	0.004	0.94 (0.467)	0.005
$R \times C \times H$	1**.88 (0.042)**	0.008	1.15 (0.310)	0.006
$M \times I \times R \times C$	1.51 (0.061)	0.012	0.95 (0.534)	0.009
$M \times I \times R \times H$	**2.94 (0.005)**	0.012	1.34 (0.176)	0.007
$M \times I \times C \times H$	1.35 (0.147)	0.011	1.09 (0.329)	0.011
$M \times R \times C \times H$	0.81 (0.724)	0.007	0.68 (0.900)	0.007
$I \times R \times C \times H$	0.88 (0.547)	0.004	1.10 (0.324)	0.005
$M \times I \times R \times C \times H$	0.91 (0.528)	0.007	1.01 (0.427)	0.010

Table S5. Two-sided analysis of variance examining effects of mineral reactivity, inoculum type, aboveground carbon inputs, presence of root exudates, and date of microcosm harvest (month 7 vs 13) on total carbon pools remaining (g per microcosm) at time of harvest. This was determined via a mass-balance approach, where total C respired from each microcosm was subtracted from total C inputs.

	**F statistic (P value)**
Mineral reactivity (M)	**15.87 (< 0.001)**
Inoculum (I)	0.01 (0.908)
Root (R) exudates	**44.18 (< 0.001)**
Aboveground carbon inputs (C)	**97.22 (< 0.001)**
Harvest (H)	**178.87 (< 0.001)**
$M \times I$	**4.92 (0.008)**
$M \times R$	**4.69 (0.011)**
$I \times R$	0.02 (0.963)
$M \times C$	1.99 (0.099)
$I \times C$	0.07 (0.929)
$R \times C$	2.84 (0.061)
$M \times H$	**3.88 (0.022)**
$I \times H$	3.88 (0.051)
$R \times H$	2.79 (0.097)
$C \times H$	**8.87 (< 0.001)**
$M \times I \times R$	0.18 (0.833)
$M \times I \times C$	1.86 (0.121)

$M \times R \times C$	0.31 (0.873)
$I \times R \times C$	0.60 (0.553)
$M \times I \times H$	1.10 (0.335)
$M \times R \times H$	0.18 (0.840)
$I \times R \times H$	1.99 (0.161)
$M \times C \times H$	0.90 (0.466)
$I \times C \times H$	2.28 (0.106)
$R \times C \times H$	0.98 (0.378)
$M \times I \times R \times C$	0.61 (0.655)
$M \times I \times R \times H$	0.82 (0.441)
$M \times I \times C \times H$	0.14 (0.967)
$M \times R \times C \times H$	1.08 (0.369)
$I \times R \times C \times H$	1.96 (0.145)
$M \times I \times R \times C \times H$	0.16 (0.960)

Table S6a. Properties of pure clay minerals used to establish a gradient of mineral reactivity in the artificial soils. Cation exchange capacity was determined on pure mineral samples at the Utah State University Analytical Laboratory, whereas surface area data are reported in Machet et al. 2011 *Applied Clay Science* 53(1) and Gao et al. 2018 *European Journal of Soil Science* 69. **b**. Composition of artificial soils used in the laboratory microcosm experiment.

a.

**Clay mineral**	**Type**	**Surface area** *$m^2 g^{-1}$*	**Cation exchange capacity** *$cmol\ kg^{-1}$*
**Kaolinite**	1:1 phyllosilicate	17.5 - 25	9.9
**Montmorillonite**	2:1 phyllosilicate	60 - 94.7	41.8
**Geothite**	Iron oxide-hydroxide	69.7 - 200	33.8

b.

**Component**	**Percentage of soil mass**		
	Low activity clay treatment	*Medium activity clay treatment*	*High activity clay treatment*
Kaolinite	10	0	0
Montmorillonite	0	10	9
Geothite	0	0	1
Quartz sand	86	86	86
Organic matter	4	4	4

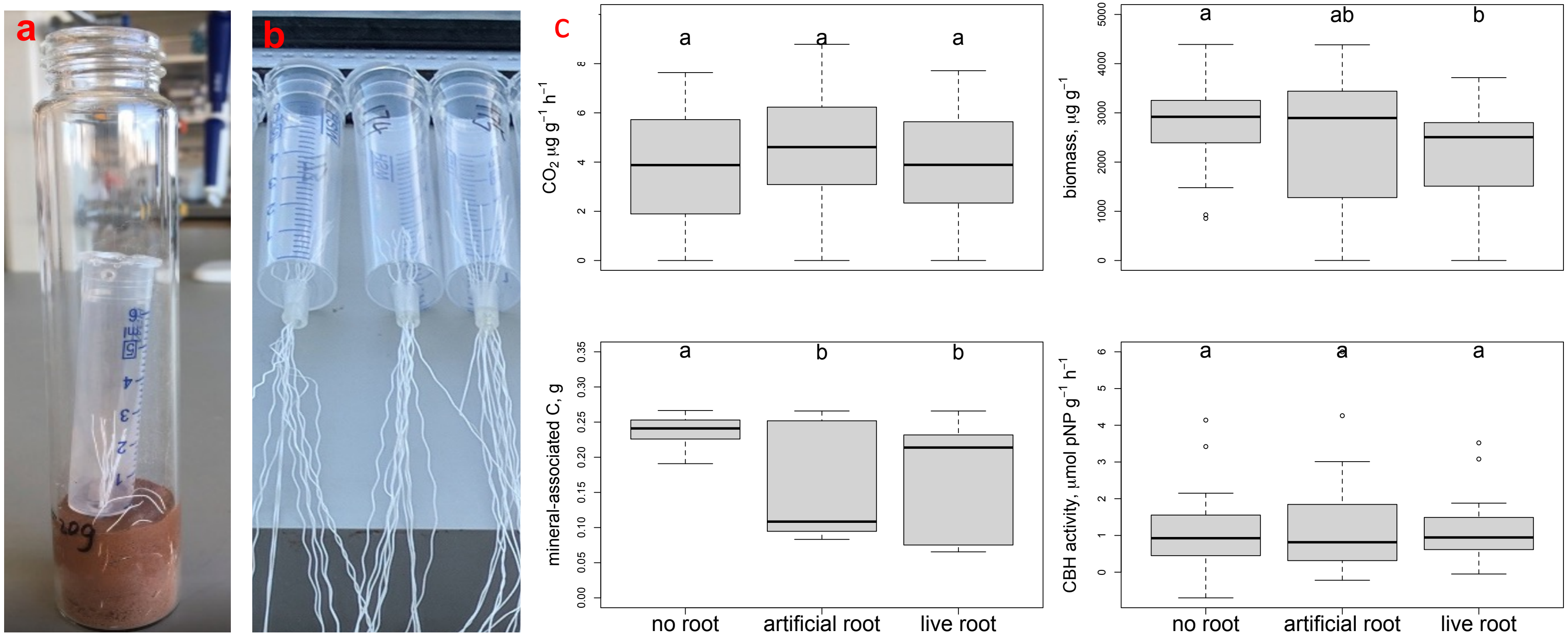

Figure S1a) Gravity-fed artificial roots constructed of hollow fiber dialysis membranes (HFMs) (Fresenius Medical Care North America, Ogden, UT) with a lumen diameter of <200 μm and wall thickness of <50 μm, shown installed in an artificial soil microcosm. Root reservoirs **(b)** were filled with artificial exudate solution every 2 weeks, over which time the solution slowly percolated into the soil. We used UV-cured Dymax glue (Dymax, Torrington, CT) to seal the tip of the reservoir and ensure fluid could only travel through the HFMs. **c.** Soil carbon pools and fluxes (CO_2 flux, microbial biomass, mineral-associated C [MAOC]) and extracellular enzyme activities were comparable in real soils exposed to live roots vs. artificial roots (N = 12 per root treatment). Letters reflect results of a Tukey's HSD post-hoc test conducted following a three-way ANOVA examining the effects of inoculum treatment (BO *vs.* FB), carbon amendment (glucose *vs.* cellulose *vs.* xylan), and root treatment (no-root control *vs.* artificial root *vs.* live root). These boxplots, as for all boxplots shown in the Supplemental Information, visualize the median and interquartile range, while the whiskers show the minimum and maximum of the data.

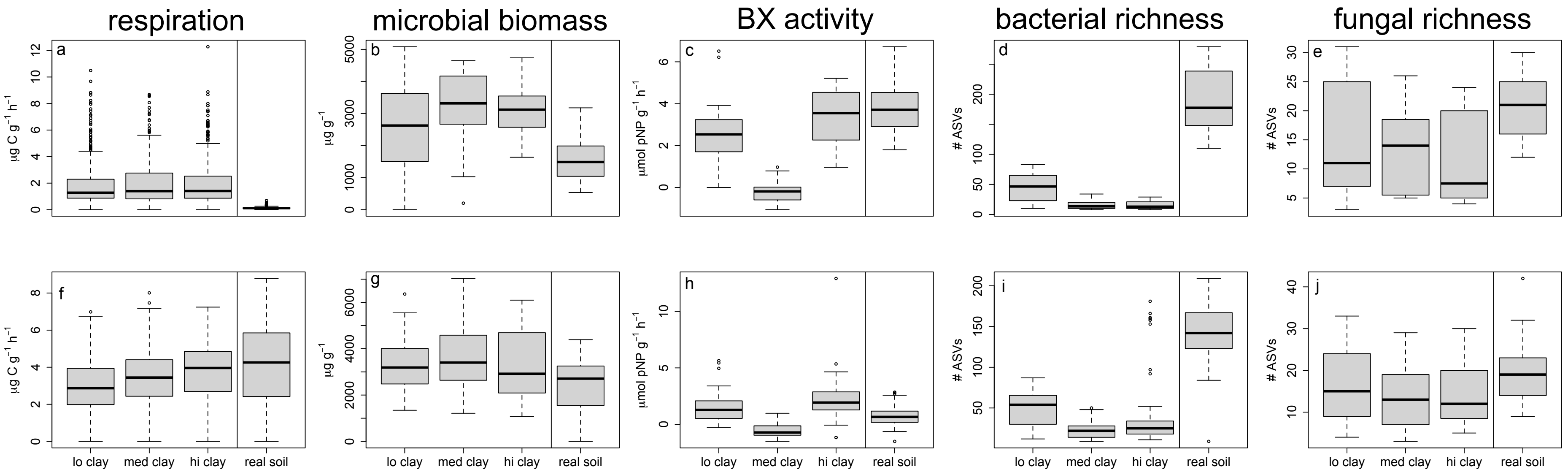

Figure S2. Biogeochemical and microbial community responses to mineral reactivity treatments (low: kaolinite, medium: montmorillonite, high: montmorillonite + goethite) in artificial soils, as compared to real (non-artificial) soils incubated under identical conditions. Artificial and real soils had similar carbon content (1.70% *vs.* 1.73%) and clay content (10% *vs.* 12.1%). Panels **a-e** show data from the first phase of the experiment (months 1-3; N = 24 per treatment). Panels **f-j** show data from the second phase (months 4-13; N = 72 per treatment). Abbreviations: BX = beta xylosidase; ASV = amplicon sequence variant

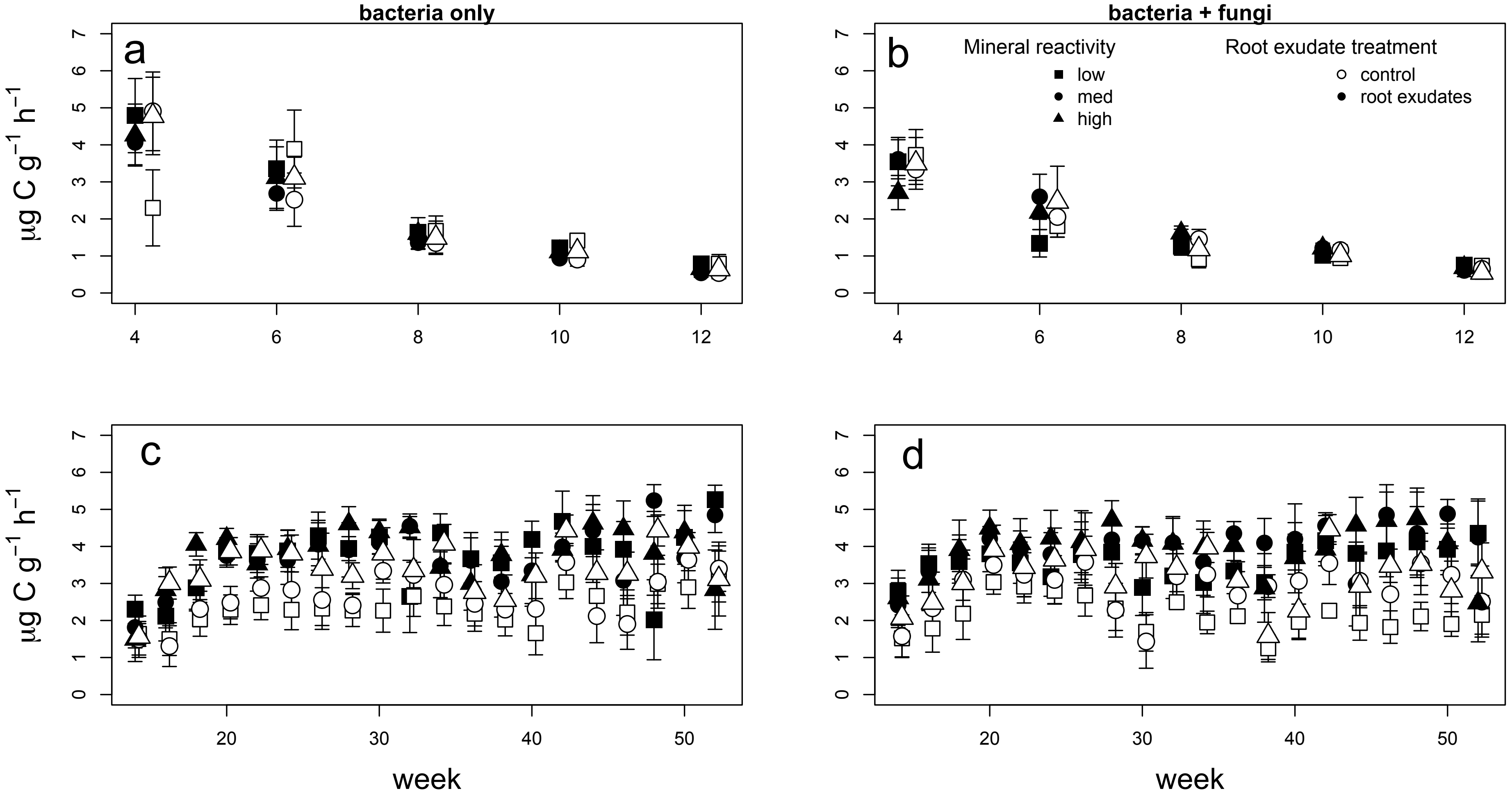

Figure S3. Mean weekly rates of CO_2 flux from artificial soils during the first (**a, b)** and second (**c, d**) experimental phases. Panels **a** and **c** show data from the BO inoculum treatment, while panels **b** and **d** display data from the FB inoculum treatment. Bars indicate standard errors; each point represents the average of 24 (panels **a, b**) or 18 microcosms (panels **c, d**) in the indicated treatment combination.

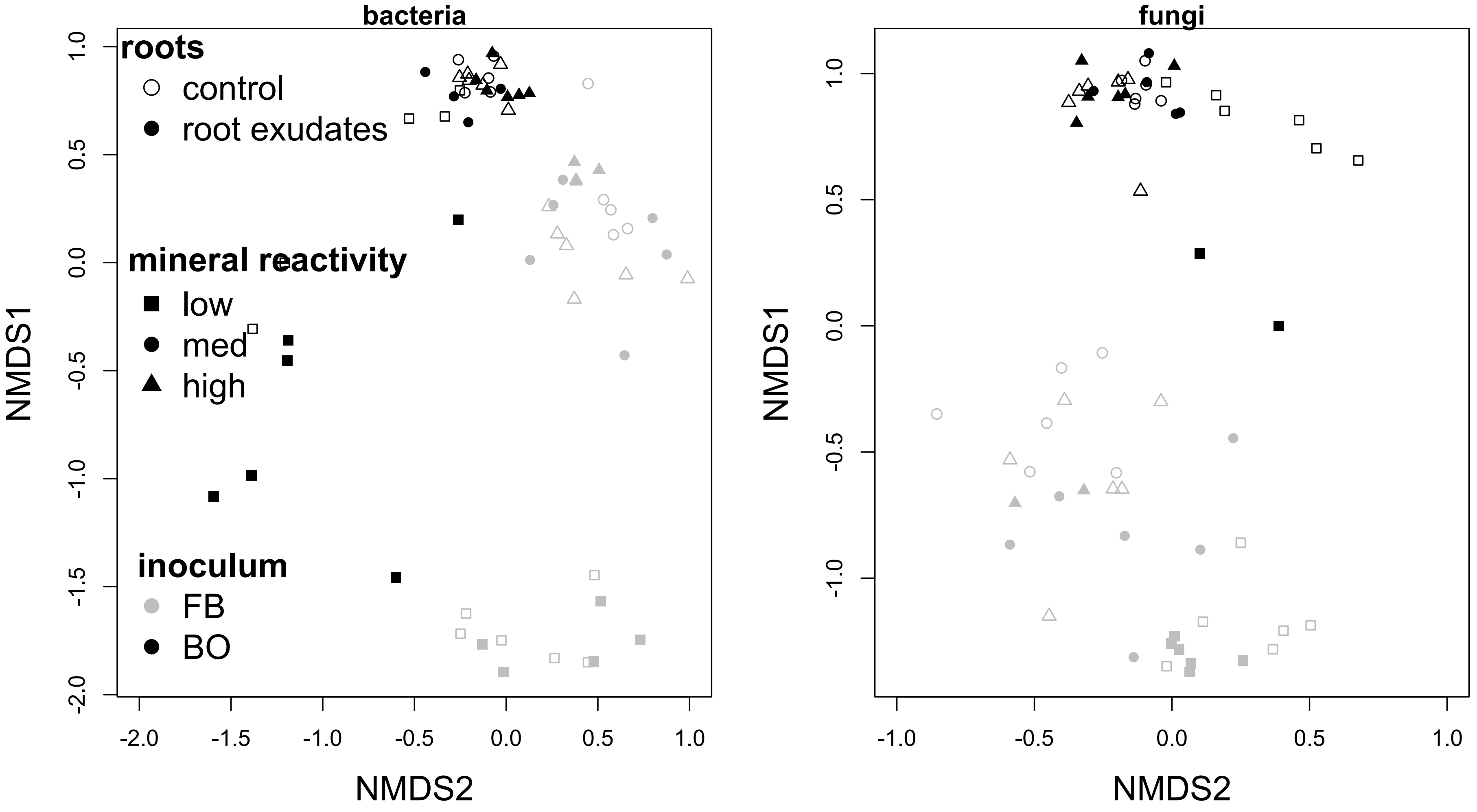

Figure S4. Non-metric multidimensional scaling analysis conducted on a Bray-Curtis dissimilarity matrix to analyse bacterial and fungal communities in artificial soils during the first phase of the experiment. Note that during this experimental interval, root exudation rates were extremely low (3.7 μg C g^{-1} d^{-1} in the first phase *vs.* 37 μg C g^{-1} d^{-1} in the second phase).

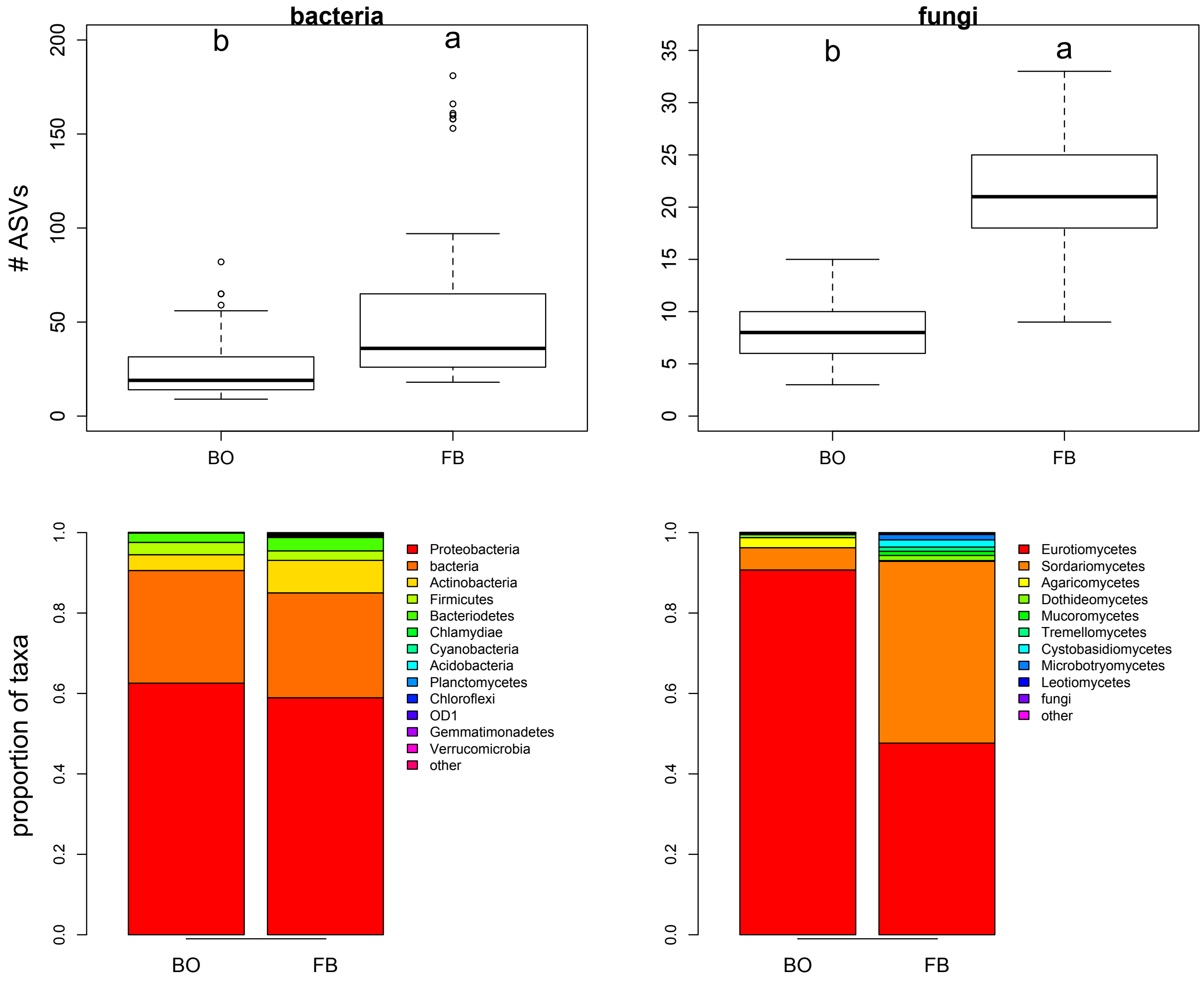

Figure S5. The top panels show the richness of bacterial and fungal 'species' (amplicon sequence variants, ASVs) observed in the bacteria-only (BO) vs. fungal and bacterial (FB) inoculum treatments. Letters show the results of a Tukey's HSD post-hoc test conducted following a five-way ANOVA. The bottom panels show the proportion of ASVs belonging to bacterial phyla and fungal classes in the two inoculum treatments. Data shown are for artificial soils only, in the second phase of the experiment (N = 108 per inoculum treatment).

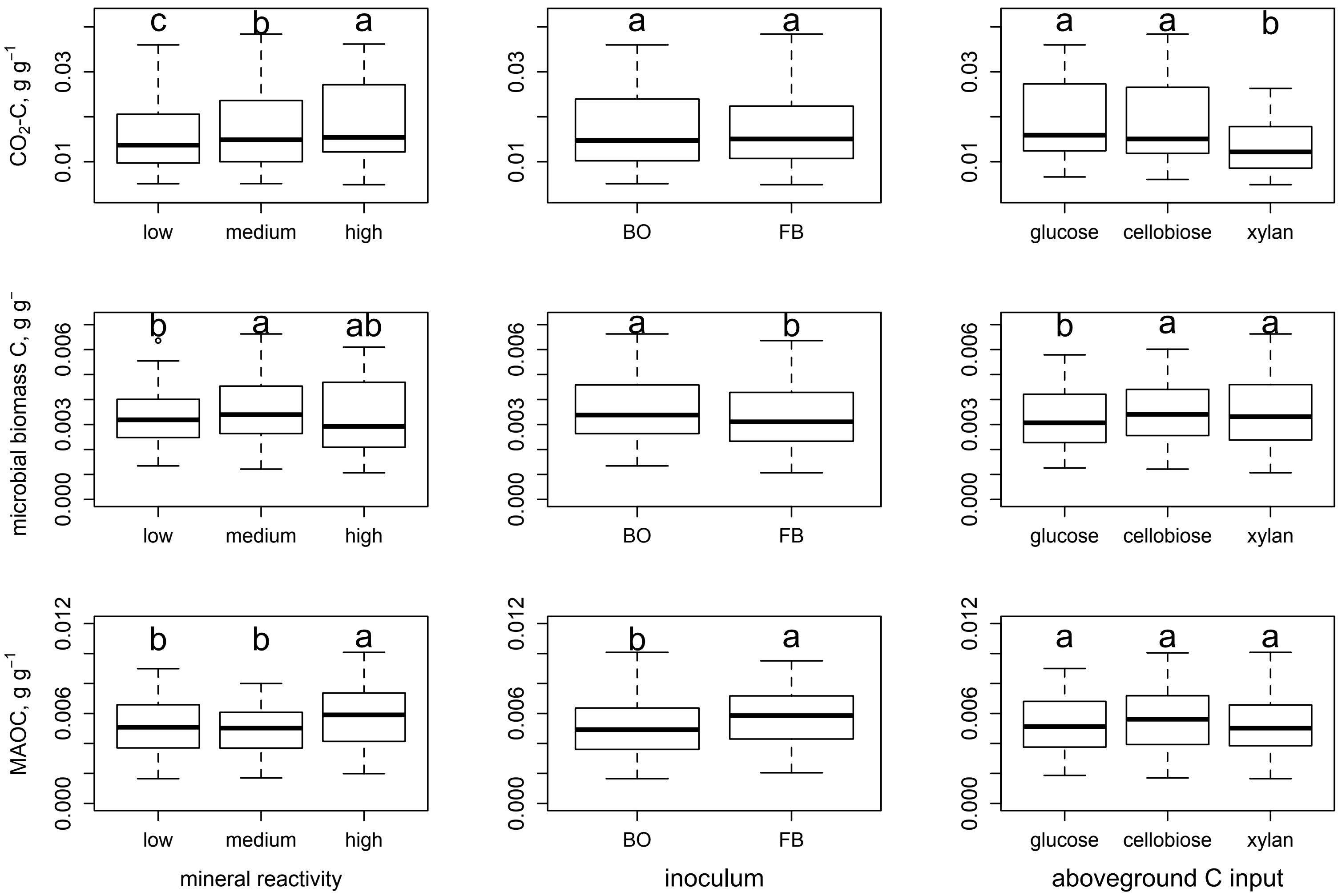

Figure S6. Effects of experimental treatments on cumulative respiration (top panels), microbial biomass (middle panels), and mineral-associated organic C [MAOC] (bottom panels) in artificial soils during the second, 10-mo phase of the experiment. Data are averaged across two harvests (at 7 months and 13 months) and root exudate treatments. Letters indicate significant differences among treatment levels using Tukey's HSD tests. N = 72 per mineral treatment, 108 per inoculum treatment, and 72 per carbon input treatment.

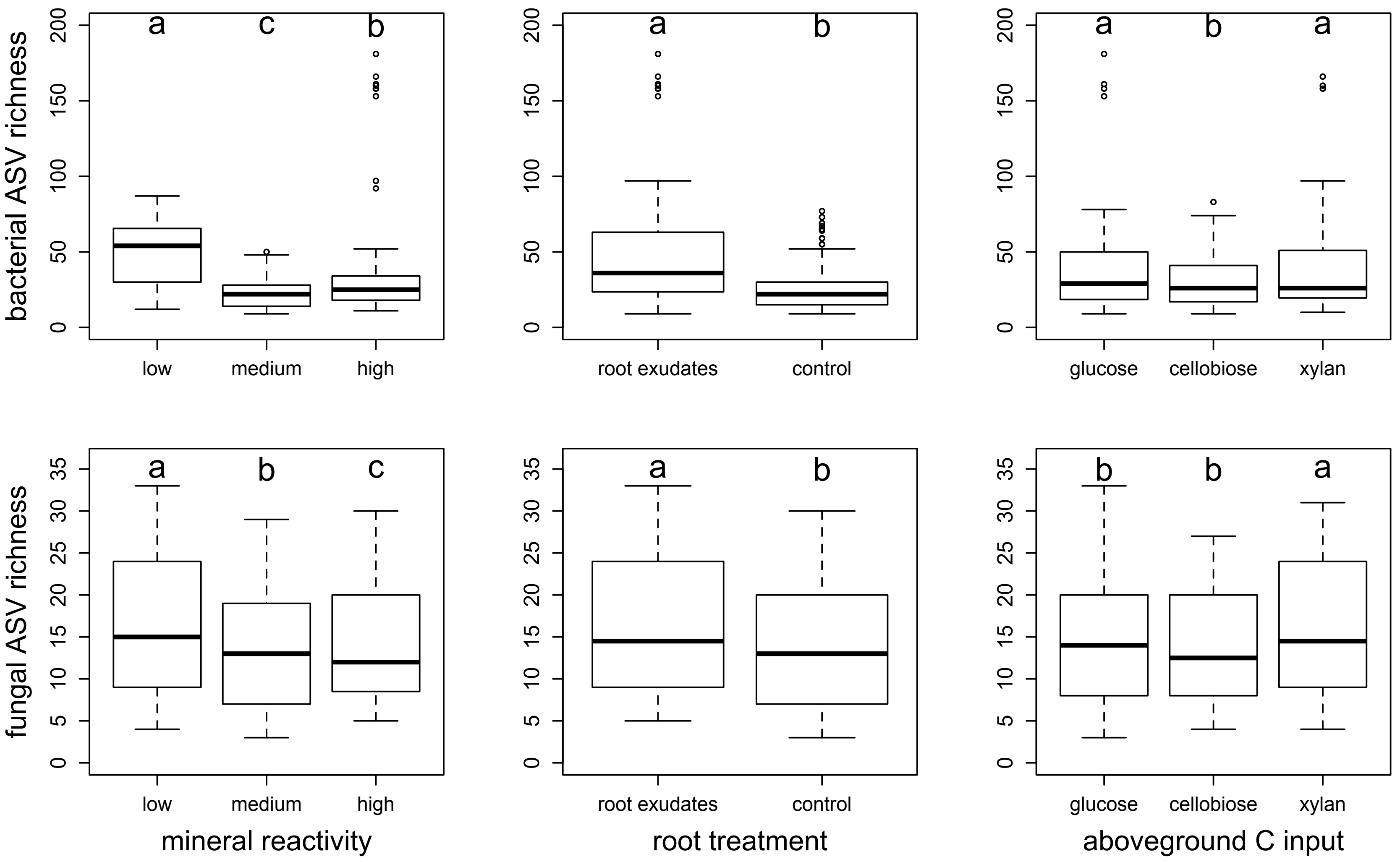

Figure S7. Effects of experimental treatments on bacterial diversity (top panels) and fungal diversity (bottom panels) in artificial soils in the second phase of the experiment. Data are averaged across harvests (7 months and 13 months). Letters indicate significant differences among treatment levels using Tukey's HSD tests. N = 72 per mineral treatment, 108 per root treatment, and 72 per carbon input treatment.

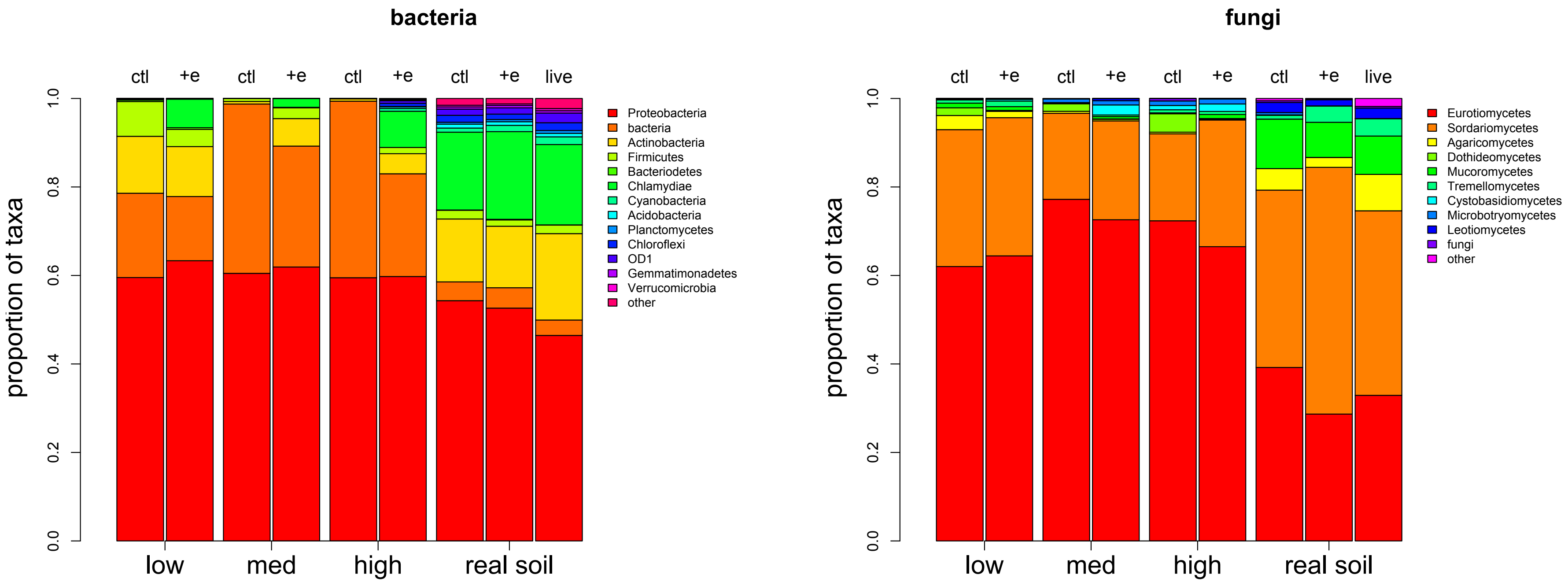

Figure S8. Proportion of ASVs belonging to bacterial phyla and fungal classes in different combinations of mineral reactivity treatments (low: kaolinite, medium: montmorillonite, high: montmorillonite + goethite) and root exudate treatments (control = ctl, root exudates = +e), as compared to real soils assigned to the same treatments (in addition to a live-root control [live]). Data are reported from the second phase of the experiment.

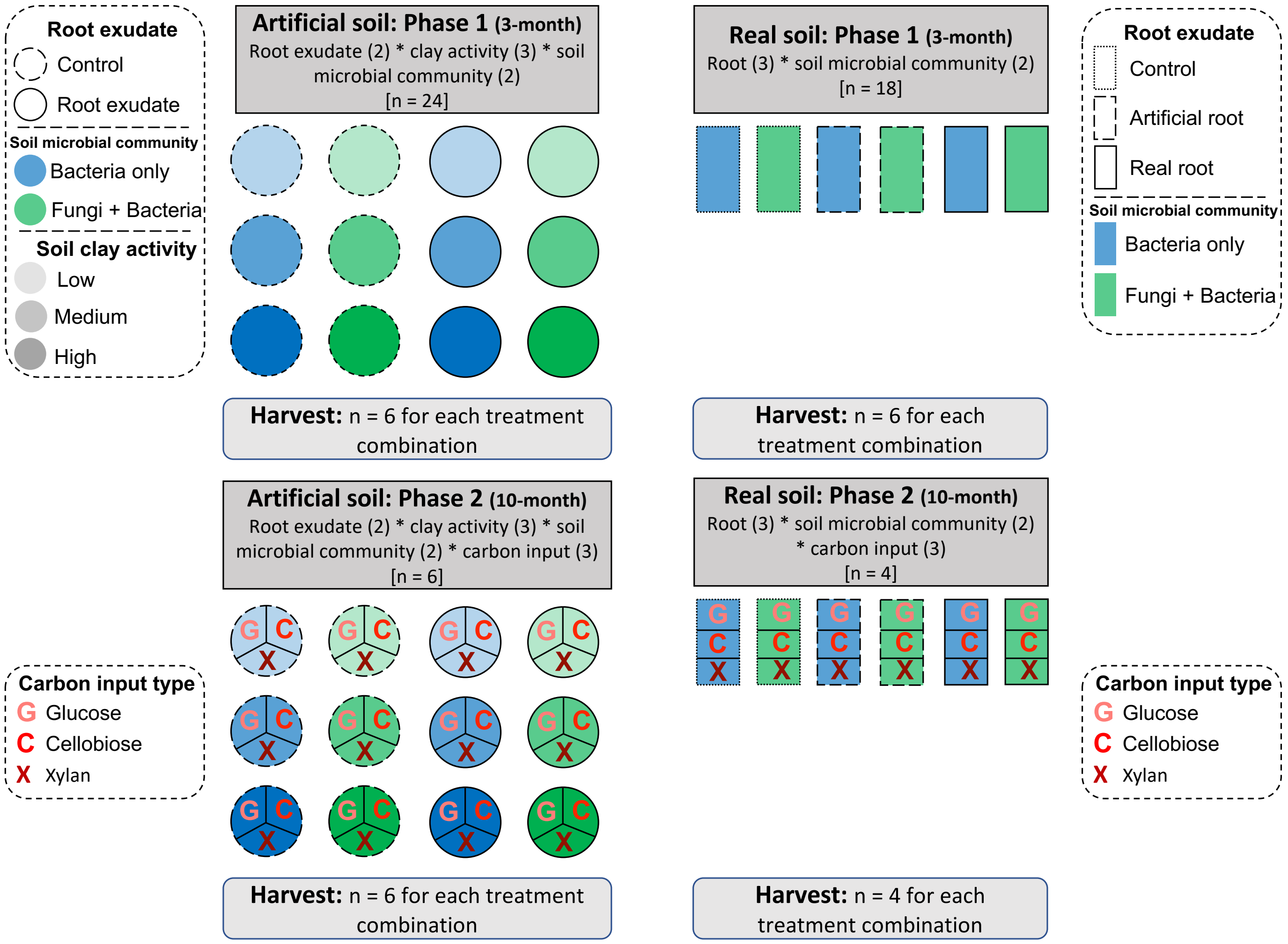

Figure S9. Schematic of all the treatments included in the microcosm experiment
