# Supplementary material for: Mineral reactivity determines root effects on soil organic carbon
Source: Nat Commun. 2023 Aug 16;14:4962. doi: 10.1038/s41467-023-40768-y (PMC10432558; doi:10.1038/s41467-023-40768-y)
Supplement: Supplementary file 3 — Reporting Summary [file 41467_2023_40768_MOESM3_ESM.pdf]

nature portfolio

Corresponding author(s): Bonnie G Waring

Last updated by author(s): Jul 27, 2023

Reporting Summary

Nature Portfolio wishes to improve the reproducibility of the work that we publish. This form provides structure for consistency and transparency in reporting. For further information on Nature Portfolio policies, see our Editorial Policies and the Editorial Policy Checklist.

Statistics

For all statistical analyses, confirm that the following items are present in the figure legend, table legend, main text, or Methods section.

n/a	Confirmed	
☐	☒	The exact sample size (*n*) for each experimental group/condition, given as a discrete number and unit of measurement
☐	☒	A statement on whether measurements were taken from distinct samples or whether the same sample was measured repeatedly
☐	☒	The statistical test(s) used AND whether they are one- or two-sided<br>*Only common tests should be described solely by name; describe more complex techniques in the Methods section.*
☐	☒	A description of all covariates tested
☐	☒	A description of any assumptions or corrections, such as tests of normality and adjustment for multiple comparisons
☐	☒	A full description of the statistical parameters including central tendency (e.g. means) or other basic estimates (e.g. regression coefficient) AND variation (e.g. standard deviation) or associated estimates of uncertainty (e.g. confidence intervals)
☐	☒	For null hypothesis testing, the test statistic (e.g. *F*, *t*, *r*) with confidence intervals, effect sizes, degrees of freedom and *P* value noted<br>*Give P values as exact values whenever suitable.*
☒	☐	For Bayesian analysis, information on the choice of priors and Markov chain Monte Carlo settings
☒	☐	For hierarchical and complex designs, identification of the appropriate level for tests and full reporting of outcomes
☒	☐	Estimates of effect sizes (e.g. Cohen's *d*, Pearson's *r*), indicating how they were calculated

Our web collection on statistics for biologists contains articles on many of the points above.

Software and code

Policy information about availability of computer code

Data collection	16S and ITS sequences were analysed with the QIIME2 pipeline (qiime2-2022.2)
Data analysis	All analyses were conducted in R version 4.1.1. (https://cran.r-project.org). Specific packages used are cited in the manuscript.

For manuscripts utilizing custom algorithms or software that are central to the research but not yet described in published literature, software must be made available to editors and reviewers. We strongly encourage code deposition in a community repository (e.g. GitHub). See the Nature Portfolio guidelines for submitting code & software for further information.

Data

Policy information about availability of data

All manuscripts must include a data availability statement. This statement should provide the following information, where applicable:

- Accession codes, unique identifiers, or web links for publicly available datasets
- A description of any restrictions on data availability
- For clinical datasets or third party data, please ensure that the statement adheres to our policy

Biogeochemical data have been uploaded to Figshare and are available at the following URLs: https://doi.org/10.6084/m9.figshare.23807352.v1; https://doi.org/10.6084/m9.figshare.23807355.v1; https://doi.org/10.6084/m9.figshare.21333048.v1. Sequence data are available at the NCBI Sequence Read Archive, http://www.ncbi.nlm.nih.gov/bioproject/1001150

Human research participants

Policy information about studies involving human research participants and Sex and Gender in Research.

Reporting on sex and gender	N/A
Population characteristics	N/A
Recruitment	N/A
Ethics oversight	N/A

Note that full information on the approval of the study protocol must also be provided in the manuscript.

Field-specific reporting

Please select the one below that is the best fit for your research. If you are not sure, read the appropriate sections before making your selection.

☐ Life sciences ☐ Behavioural & social sciences ☒ Ecological, evolutionary & environmental sciences

For a reference copy of the document with all sections, see nature.com/documents/nr-reporting-summary-flat.pdf

Ecological, evolutionary & environmental sciences study design

All studies must disclose on these points even when the disclosure is negative.

Study description	We constructed artificial root-soil systems to test hypotheses about controls on soil carbon formation and loss. The experiment was a fully factorial manipulation of four factors: soil mineralogy, presence of roots, chemistry of soil carbon inputs, and composition of the soil microbial community.
Research sample	The experimental unit in this study was an artificial root-soil system in an individual laboratory microcosm, which is intended to represent a randomly chosen sample from a population of soil profiles (pedons). We quantified biogeochemical variables for 288 artificial soil microboms and 108 'real' soil microcosms as an experimental control, so the total sample size was N = 396 (Please see Fig S9 for a schematic). Sample sizes were determined with a preliminary power analysis.
Sampling strategy	We conducted preliminary power analyses to identify the minimum number of microcosms in each treament group that would be needed to have sufficient power to detect a 10% effect size.
Data collection	Data were collected by Guopeng Liang, supervised by Bonnie Waring and John Stark. All raw data - i.e. instrument files from the Shimadzu gas chromatograph, Picarro Isotope Analyzer, Costech 4010 Elemental Analyzer, and microplate spectrophotometer - were immediately uploaded to the cloud (via Box) and reviewed by all co-authors.
Timing and spatial scale	The experiment ran for 13 months, with CO2 sampled at regular intervals and destructive harvests at 3, 7, and 13 months (evenly spaced across the incubation). These time intervals were chosen based upon preliminary experiments in which we monitored changes in C cycling in artificial soils, and determined the time frames over which significant biogeochemical changes took place. The experiment commenced in May 2020 and ended in June 2021. CO2 sampling and harvests were performed at the scale of individual soil microcosms.
Data exclusions	No data were excluded
Reproducibility	We conducted the same experiments in artificial and real soils to assess whether patterns were similar. The artificial soil microcosms (N = 288) and the 'real' soil microcosms (N = 108) were incubated and sampled at the same time, and results of this comparison are reported in the manuscript. We did not, however, repeat or reproduce the experiment with additional artificial or real soils - all data were collected between May 2020 and June 2021.
Randomization	Microcosms were randomly assigned to treatments. Samples collected from the microcosms were analyzed in random order with respect to treatment.
Blinding	Although we did not formally blind experimental units, each microcosm was assigned a number (from 1 - 396) and this was the only identifier used during data collection and processing. In other words, the experimenters did not know to which treatment group a given microcosm belonged while it was being sampled, as the label was non-informative with respect to treatment.

Did the study involve field work? ☐ Yes ☒ No

March 2021

Reporting for specific materials, systems and methods

We require information from authors about some types of materials, experimental systems and methods used in many studies. Here, indicate whether each material, system or method listed is relevant to your study. If you are not sure if a list item applies to your research, read the appropriate section before selecting a response.

Materials & experimental systems

n/a	Involved in the study	
☒	☐	Antibodies
☒	☐	Eukaryotic cell lines
☒	☐	Palaeontology and archaeology
☒	☐	Animals and other organisms
☒	☐	Clinical data
☒	☐	Dual use research of concern

Methods

n/a	Involved in the study	
☒	☐	ChIP-seq
☒	☐	Flow cytometry
☒	☐	MRI-based neuroimaging
